# Draft Genome Sequences of Three Nontypeable Strains of *Haemophilus influenzae*, C188, R535, and 1200, Isolated from Different Types of Disease

**DOI:** 10.1128/genomeA.00035-17

**Published:** 2017-03-23

**Authors:** Ulrike Kappler, Rabeb Dhouib, Remya Purushothaman Nair, Alastair G. McEwan

**Affiliations:** School of Chemistry & Molecular Biosciences, Australian Infectious Diseases Centre, The University of Queensland, St. Lucia, Queensland, Australia

## Abstract

Nontypeable *Haemophilus influenzae* is a persistent human respiratory pathogen known to be involved in a range of acute and chronic respiratory diseases. Here, we report the genome sequences of three *H. influenzae* strains isolated from sputum, otitis media, and blood. Comparative analyses revealed significant differences in the gene contents including the presence of genes mediating antibiotic resistance.

## GENOME ANNOUNCEMENT

*Haemophilus influenzae* (HI) is a host adapted human pathogen that usually resides in the respiratory tract where it can either exist as a commensal or cause and contribute to acute or chronic diseases such as otitis media, sinusitis, conjunctivitis, chronic obstructive pulmonary disease (COPD), asthma, and bronchiectasis (1). Recently, an increase in respiratory disease cases caused by nontypeable HI (NTHi) strains has been noted, and this includes more aggressive forms of disease that were previously not thought to be associated with NTHi (1–3).

Despite having small genome sizes (1.7 to 1.9 Mb; ~1,700 to 1,900 genes) which is in keeping with the strong specialization of *H. influenzae* to exclusive growth in the human host, *H. influenzae* strains are known to be genetically variable, with only about 1,400 to 1,500 genes being common to strains (4, 5), and significant numbers of unique genes being found in each strain.

Here, we have sequenced the genomes of NTHi strains isolated from different types of disease: strain C188 is a blood isolate, NTHi 1200 originates from a Finnish study of otitis media, and R535 is a sputum isolate (http://pubmlst.org/hinfluenzae/, accessed 10 January 2017). All three strains have been used in previous studies mostly as parts of strain collections (6, 7), and the availability of complete genome data for these strains will enhance the ability to compare and fully interpret previous and future work (Table 1).

**TABLE 1:** Properties of the draft genomes for *H. influenzae* strains C188, R535, and 1200

Strain name	Bioproject sample no.	Accession no.	Coverage	No. of contigs	Completeness^a^	Size (Mb)	G+C content (%)	No. of genes	No. of proteins
C188	SAMN05942198	MQMI00000000	455×	56	99.45 (0.07)	1.85	37.9	1,872	1,719
R535	SAMN05938535	MQMJ00000000	545×	61	99.35 (0.23)	1.77	37.9	1,774	1,623
1200	SAMN05942090	MQMH00000000	682×	64	98.86 (0)	1.88	38	1,906	1,743

^a^ As determined by CHECKM (13). In parentheses: level of contamination, which is negligible for all three genomes.

Genomic DNA was isolated using the PureLink genomic DNA kit (Life Technologies, Inc.) and adjusted to 5 ng/mL using a Qubit broad range assay (Thermo Fisher Scientific) before sequencing at the Australian Centre for Ecogenomics (ACE) using the manufacturer’s standard protocol for Nextera XT libraries (Illumina). Libraries were pooled at equimolar amounts after quantification with HS D500 Agilent tape (Agilent, TapeStation). Sequencing was performed on NextSeq 500 (Illumina) on a 2 × 150 PE run with V2 chemistry at a depth of 0.5 Gb per sample. Assembly used Spades v3.9.0 (8) with default parameters for isolate genomes. Read mapping used BamM v1.7.3 (https://github.com/Ecogenomics/BamM) which includes SAMtools (9). Annotation used the NCBI annotation pipeline (NCBI_PGAP) (10).

As expected, the three genomes show the typical variation in the number of protein encoding genes: 1,719 (C188), 1,623 (R535), and 1,743 (1200). A proteinortho (11) comparison of the protein coding genes in *H. influenzae* RD (12) and the three newly sequenced strains revealed that only 1,356 proteins were common to all four strains. Varying numbers of unique proteins (C188: 132; R535: 29; 1200: 136) were present, and these were dominated by hypothetical proteins in all cases (52 to 74%). In addition, unique proteins of phage/transposon origin were particularly abundant in NtHi strain 1200, which also appeared to carry several genes encoding antimicrobial resistance that were notably absent in the other strains, which both originate from an earlier isolation period as well as geographically distinct locations.

### Accession number(s).

The whole-genome shotgun projects have been deposited in GenBank under the accession numbers specified in Table 1. The versions described in this paper are the first versions.
